# Knowledge of HIV/AIDS transmission modes and attitudes toward HIV/AIDS infected people and the level of HIV/AIDS awareness among the general population in the kingdom of Saudi Arabia: A cross-sectional study

**DOI:** 10.3389/fpubh.2022.955458

**Published:** 2022-09-27

**Authors:** Fadi S. Qashqari, Radi T. Alsafi, Saeed M. Kabrah, Rayda'a A. AlGary, Sara A. Naeem, Malak S. Alsulami, Hatim Makhdoom

**Affiliations:** ^1^Microbiology Department, Faculty of Medicine, Umm Al-Qura University, Makkah, Saudi Arabia; ^2^Laboratory Medicine Department, Faculty of Applied Medical Sciences, Umm Al-Qura University, Makkah, Saudi Arabia; ^3^Applied Medical Sciences College, Laboratory Technology Department, Taibah University, Almadinah Almunwarah, Saudi Arabia

**Keywords:** attitude, HIV/AIDS, knowledge, Saudi Arabia, transmission modes

## Abstract

**Introduction:**

The human immunodeficiency virus (HIV) and acquired immunodeficiency syndrome (HIV/AIDS) are worldwide public health issues. Since Saudi Arabia is growing more accessible to the outside world, it is critical to analyze the general population's knowledge of HIV/AIDS transmission modes and attitudes toward HIV/AIDS infected people, and the level of HIV/AIDS awareness. Therefore, this study aimed to assess the knowledge of HIV/AIDS transmission modes and attitudes toward HIV/AIDS infected people, as well as the level of HIV/AIDS awareness among the general population in the Kingdom of Saudi Arabia.

**Methods:**

The current online community-based cross-sectional descriptive study was conducted among the general population of the Kingdom of Saudi Arabia using a self-administrated electronic questionnaire between October 2017 and February 2018. A score <3 was considered a negative response. In contrast, scores of 3 and 4 were considered positive responses. The sum score of each outcome was evaluated according to Bloom's cutoff point. The scores for knowledge and attitude were transformed into mean percentage scores by dividing the sum scores obtained by the respondents by the number of items multiplied by 100. Consequently, the overall mean percentage of scores for each category of knowledge and attitude at 60% and above was considered a good level, whereas <60% was deemed a poor level.

**Results:**

A total of 2,081 subjects residing in the Kingdom of Saudi Arabia participated in this survey. The mean score of the participant's responses to knowledge items on HIV/AIDS transmission modes was 84.2 ± 15.8%. The mean score of the participant's responses to attitude items toward HIV/AIDS infected people was 50.1 ± 49.9%. The mean score of the participant's responses to attitude items toward the level of HIV/AIDS awareness in Saudi Arabia was 55.7 ± 3.6%. Many socio-demographic variables were significantly associated with mean scores of knowledge of HIV/AIDS transmission modes, mean scores of attitude toward HIV/AIDS infected people, and mean scores of attitude toward the level of HIV/AIDS awareness (*P-*value ≤ 0.05). The Spearman rank correlation explained significant negative linear correlations between knowledge of HIV/AIDS transmission modes-attitudes toward HIV/AIDS infected people and knowledge of HIV/AIDS transmission modes-attitudes toward the level of HIV/AIDS awareness of r = −0.040 and r = −0.070, respectively (*P*-value ≤ 0.05). Otherwise, there was a positive linear correlation between attitudes toward HIV/AIDS infected people-attitudes toward the level of HIV/AIDS awareness (r = 0.080, *P*-value = 0.072).

**Conclusions:**

The study showed that a high percentage of the respondents had good knowledge of HIV/AIDS transmission modes. However, a low rate of the study respondents had positive attitudes toward HIV/AIDS infected people and the level of HIV/AIDS awareness among the general population in Saudi Arabia. Therefore, there is a dire need for advocacy campaigns and policies to help reduce HIV stigmatization levels.

## Introduction

Infection with the Human Immunodeficiency Virus (HIV) and the Acquired Immune Deficiency Syndrome (AIDS) continues to be a significant public health issue (1, 2). Even though the number of HIV cases in the Middle East and North Africa (MENA) region is low compared to other areas, recent research has revealed that HIV incidence is rising, particularly among high-risk populations (3). According to the most recent figures on the condition of the AIDS epidemic (2).

Because of social, cultural, and religious taboos, accurate data on HIV/ the prevalence and incidence of HIV/AIDS has been slow in many Middle Eastern nations compared to other regions (4). Nevertheless, data in some countries is inadequate, and there is significant variation within the region. Because HIV remains a contentious issue in the MENA region, data on its prevalence and trends are poor, under-reporting is likely, and it is impossible to collect precise figures or determine the specific causes of HIV levels and trends (5).

Understanding people's knowledge and attitudes about HIV/AIDS are one of the cornerstones of fighting the disease. The most common problem people living with HIV/AIDS (PLWHA) face is the general public's lack of knowledge or miseducation about the disease (6); this, in turn, leads to or precipitates discrimination against them, which leads to or precipitates problems with disclosure, social isolation, access to antiretroviral therapy, and psychological support (7, 8).

Furthermore, several factors are linked to HIV knowledge and play a significant influence in influencing the amount of HIV stigma. These determinants are primarily education and HIV attitudes. Increased HIV education and awareness are two excellent techniques for reducing stigma, among many others. As a result, as one's level of education rises, so does one's understanding of HIV, unfavorable views about PLWHA fall, and HIV stigma falls (9, 10).

The theory of planned behavior can be utilized as a framework for creating and executing educational interventions to prevent HIV/AIDS among addicts due to the intervention's strong impact on its structural components (11). Moreover, in high school female students, educational interventions based on the Health Belief Model were successful in promoting AIDS preventive beliefs by increasing knowledge and perceived susceptibility (12).

Several quasi-experimental studies that looked at the impact of educational and awareness interventions on HIV stigma back this up (13, 14). For example, a study in Canada found that raising participants' HIV awareness helped them become community influence champions, which reduced HIV stigma (13). Furthermore, a more sensitive cultural study found that the authors' educational and awareness interventions in a healthcare context reduced HIV stigma and prejudice in Egypt (14). Finally, the majority of the US Centers for Disease Control and Prevention (CDC) activities are centered on programs that combine public education and social marketing campaigns (15).

In Saudi Arabia, the rate of HIV infection increased by 0.5–2.5 percent between 1984 and 2009 (16). HIV infections are more common in Jeddah than in any other city in the Kingdom, which could be attributed, at least in part, to the fact that Jeddah is the country's principal seaport and airport, with higher population movement (17). While immigrants account for most HIV cases in Saudi Arabia, there has been a considerable increase in the proportion of cases among the Saudi population (18).

Even though the introduction of highly active antiretroviral medication has reduced mortality and improved quality of life, HIV remains a contentious topic and taboo in the MENA region (19). The region's data continues to reveal a low level of understanding, which is linked to a higher level of stigma (20, 21). A study of university students in the United Arab Emirates, for example, found serious knowledge gaps as well as high levels of fear and intolerance toward PLWHA (22). The study surveyed medical students at Qassim University in Saudi Arabia showed a modest level of knowledge and negative attitudes toward PLHIV (23).

Based on the preceding, it is critical to have a comprehensive understanding of the degree of knowledge and attitudes about HIV/AIDS so that suitable and relevant awareness and prevention programs may be planned. As a result, the goal of this study was to assess the knowledge of HIV/AIDS transmission modes and attitudes toward HIV/AIDS infected people, as well as the level of HIV/AIDS awareness among the general population in the Kingdom of Saudi Arabia.

## Methods

### Study design, study site, and study period

The current online community-based cross-sectional descriptive study was conducted using a self-administrated electronic questionnaire to assess the Saudi population's knowledge regarding transmission modes of HIV/AIDS and their attitude toward HIV/AIDS-infected people. This questionnaire was distributed among the general population of the Kingdom of Saudi Arabia between October 2017 and February 2018.

### Study tool and data collection technique

A pre-tested structured, standard questionnaire consisting of 36 close-ended questions was randomly administered to the general population of the Kingdom of Saudi Arabia *via* social media platforms such as WhatsApp, Twitter, Facebook, Instagram, etc. Additionally, it was distributed in the public campaign regarding World AIDS Day in Heraa General Hospital.

The questionnaires were designed based on the AIDS Indicator Survey model developed by the MEASURE DHS program and the AIDS survey model with some indicators from the National HIV/AIDS prevention programs for young people (24). Five specialists checked the questionnaire's content validity in the fields of public health, epidemiology, and biostatistics. An online pilot study of 45 participants was conducted to ensure the survey's acceptability and consistency. Then, minor modifications were made according to the pilot study results.

The questionnaire was divided into four main sections: (1) socio-demographic characteristics (6 items), (2) knowledge of HIV/AIDS transmission modes (12 items), (3) attitude questions toward HIV/AIDS infected people (7 items), and (4) attitude questions toward the level of HIV/AIDS awareness in Saudi Arabia (11 items).

### Inclusion criteria

Adults aged 16 years or older (both genders) residing in Saudi Arabia and signed consent forms were included in the present study. To ensure that participants were still living in Saudi Arabia, they were asked to provide their housing address and the name of the neighborhood.

### Exclusion criteria

People less than 16 years or from a nation other than Saudi Arabia were excluded from the present study.

### Sample size

The sample size for this study was calculated to be a total of 384 individuals living in Saudi Arabia aged 18 and over, including Saudi and non-Saudi. This sample size was estimated as described by Kadam and Bhalerao (25), based on a confidence interval of 95% and 5% marginal error with a 0.05 alpha level (25).

### Ethical considerations

The participants were asked to approve their participation before proceeding with the online survey. Ethical approval was obtained from the Faculty of Applied Medical Sciences ethics committee. Informed consent for an internet survey was also obtained from each participant. No monetary rewards were given for completing the questionnaire.

### Statistical analysis

The Statistical Package for Social Science (IBM SPSS) version 20 was used for data analysis. Findings were presented as frequency and percentage tables; a Chi-square test was performed to identify relationships for categorical variables with *P-*value ≤ 0.05. The One-Way ANOVA test determined the mean differences in quantitative variables between the two groups. Meanwhile, Spearman's rank correlation coefficient (*P-*value ≤ 0.05) was applied to assess the association between knowledge and attitudes.

The measurement scale of knowledge and attitude questions was used on a five-point Likert scale (5 = Always true, 4 = Sometimes true, 3 = neutral, 2 = sometimes but infrequently true, and 1 = Never true) and (5 = Strongly Agree, 4 = agree, 3 = neutral, 2 = disagree, and 1 = strongly disagree), respectively, then changed and presented in this paper as a dichotomous classification. A score less than three was considered a negative response. In contrast, scores of 3 and 4 were considered positive responses. For the responses of participants to attitude items toward the level of HIV/AIDS awareness in Saudi Arabia, we kept the scale in its original form (5 = strongly agree, 4 = agree, 3 = neutral, 2 = disagree, and 1 = strongly disagree).

The sum score of each outcome was evaluated according to Bloom's cutoff point. The scores for knowledge and attitude were transformed into mean percentage scores by dividing the sum scores obtained by the respondents by the number of items multiplied by 100. Consequently, the overall mean percentage of scores for each category of knowledge and attitude at 60% and above was considered a good level, whereas <60% was deemed a poor level (26).

## Results

A total of 2,081 subjects residing in the Kingdom of Saudi Arabia participated in this survey. More than half of the participants (59.0%) had ages ranging between 20 and 29 years. Almost three-quarters (73.2%) of the study participants were females. The vast majority of the participants were Saudi people (94.3%). The marital status of more than half of the study participants (57.0%) was single. 67.6% of the study participants had a bachelor's degree. The monthly income of 68% of the study participants was ≤ 8,699 Saudi Arabian Riyals (Table 1).

**Table 1:** Socio-demographic characteristics (*N* = 2,081).

**Variable**	**Response option**	***N***	**%**
Age (Year)	<20	145	7.0
	20–29	1,229	59.0
	30–39	420	20.2
	≥40	287	13.8
Gender	Male	558	26.8
	Female	1,523	73.2
Nationality	Saudi	1,963	94.3
	Non-Saudi	118	5.7
Marital status	Single	1,187	57.0
	Married	817	39.3
	Divorced	62	3.0
	Widowed	15	0.7
Education level	Uneducated	1	0.0
	Elementary	0	0.0
	Intermediate	15	0.7
	High school	317	15.3
	Diploma	110	5.3
	Bachelor	1,403	67.6
	Higher diploma	23	1.1
	Masters	154	7.4
	PhD	54	2.6
Monthly income (Saudi	≤8699	1,415	68.0
arabian riyal)	8,700–11,999	266	12.8
	12,000–15,299	179	8.6
	15,300–20,159	137	6.6
	≥20,160	84	4.0

Twelve items assessed the knowledge of HIV/AIDS transmission modes among the study participants. The highest level of good knowledge was for the item on HIV/AIDS general knowledge. In contrast, the lowest level of good knowledge was evident in responses to items related to HIV infection cases in Saudi Arabia. The mean score of the participant's responses to knowledge items regarding HIV/AIDS transmission modes was 84.2 ± 15.8% (Table 2).

**Table 2:** Responses of participants to knowledge items on HIV/AIDS transmission modes (*N* = 2,081).

**Knowledge items**	**Yes *n* (%)**	**No** ** *n* (%)**
Do you know what HIV/AIDS is?	1,899 (91.3)	182 (8.7)
Is HIV infectious?	1,839 (88.4)	242 (11.6)
Are there cases of HIV infection in Saudi Arabia?	1,608 (77.3)	473 (22.7)
Does HIV transmit through coughing or sneezing?	1,794 (86.2)	107 (13.8)
Does HIV transmit through sharing food utensils with an infected person?	1,746 (83.9)	335 (16.1)
Does HIV transmit through touching the blood of an infected person?	1,752 (84.2)	329 (15.8)
Can HIV be transmitted through a mosquito bite?	1,700 (81.7)	381 (18.3)
Can HIV be transmitted through cats and dogs?	1,725 (82.9)	356 (17.1)
If the mother has HIV, the risk of transmission during pregnancy and breastfeeding is possible?	1,660 (79.8)	421 (20.2)
Does HIV transmit through sexual relations with an infected person more than 90% of the time?	1,798 (86.4)	283 (13.6)
Is a person living with HIV in Saudi Arabia isolated or imprisoned?	1,773 (85.2)	308 (14.8)
Is there a treatment for patients with HIV similar to that for other diseases?	1,729 (83.1)	352 (16.9)

The attitude toward HIV/AIDS-infected people among the study participants was evaluated using seven items. The highest level of positive attitude was for the item supporting the dissemination of awareness about how HIV is transmitted. In contrast, the lowest level of positive attitude was evident in responses to items related to accepting the idea of marrying an HIV-infected person. The mean score of the participant's responses to attitude items toward HIV/AIDS infected people was 50.1 ± 49.9% (Table 3).

**Table 3:** Responses of participants to attitude items toward HIV/AIDS infected people (*N* = 2,081).

**Attitude items**	**Yes *n* (%)**	**No** ** *n* (%)**
Do you accept the idea of marrying an HIV-infected person?	113 (5.4)	1,962 (94.6)
Do you accept living with an HIV-infected person?	689 (33.2)	1,386 (66.8)
Do you accept the idea of complete isolation of people living with HIV?	928 (44.7)	1,150 (55.3)
Do you accept taking care of an HIV-infected person?	1,515 (73.1)	558 (26.9)
Do you support informing the official authorities of someone confirmed to have HIV?	1,716 (83.1)	349 (16.9)
Do you support the prevention of HIV patients from completing their studies/work?	246 (11.9)	1,829 (88.1)
Do you support the dissemination of awareness about how HIV is transmitted?	2,058 (99.1)	18 (0.9)

Almost 80.6% rated with strong agreement that “it is better to educate high school students and universities,” while 18.8% strongly disagreed with the question being asked as “the level of HIV awareness in Saudi Arabia is sufficient”. The mean score of the participant's responses to attitude items toward the level of HIV/AIDS awareness in Saudi Arabia was 55.7 ± 3.6% (Table 4).

**Table 4:** Responses of participants to attitude items toward the level of HIV/AIDS awareness in Saudi Arabia (*N* = 2081).

**Variable**	**Response option** ***n*** **(%)**				
	**Strongly agree**	**Agree**	**Neutral**	**Disagree**	**Strongly disagree**
The level of HIV awareness in Saudi Arabia is sufficient	153 (7.4)	197 (9.5)	676 (32.6)	655 (31.6)	390 (18.8)
A culture of awareness about how the virus is transmitted should be disseminated	1,583 (76.3)	341 (16.4)	95 (4.6)	23 (1.1)	34 (1.6)
Saudi society needs more awareness about the virus and its transmission modes	1,598 (77.0)	326 (15.7)	90 (4.3)	22 (1.1)	38 (1.8)
It is better to educate high school students and universities	1,671 (80.6)	268 (12.9)	77 (3.7)	24 (1.2)	33 (1.6)
I prefer the awareness to be *via* social media	1,355 (65.4)	447 (21.6)	191 (9.2)	47 (2.3)	33 (1.6)
I prefer the awareness to be through lectures and awareness campaigns	1,102 (53.2)	500 (24.1)	336 (16.2)	89 (4.3)	44 (2.1)
I prefer the awareness to be *via* radio and television	845 (40.8)	476 (23.0)	485 (23.4)	193 (9.3)	73 (3.5)
Dissemination of the culture of awareness about HIV transmission is the responsibility of Saudi society	983 (47.5)	506 (24.4)	416 (20.1)	117 (5.6)	49 (2.4)
Dissemination of the culture of awareness about HIV transmission is the responsibility of the ministry of education	936 (45.3)	569 (27.5)	409 (19.8)	97 (4.7)	56 (2.7)
Dissemination of the culture of awareness about HIV transmission is the responsibility of the media	1,027 (49.7)	535 (25.9)	353 (17.1)	102 (4.9)	49 (2.4)
Dissemination of the culture of awareness about HIV transmission is the responsibility of the ministry of health	1,444 (69.8)	388 (18.8)	172 (8.3)	30 (1.5)	34 (1.6)

Among study participants' characteristics variables, age, nationality, marital status, education level, and monthly income were significantly associated with mean scores of knowledge of HIV/AIDS transmission modes (*P-*value ≤ 0.05). Furthermore, a significant difference was found in age, education level, and monthly income with mean scores of attitude toward HIV/AIDS infected people (*P*-value ≤ 0.05). Furthermore, age, nationality, marital status, and education level have a significant relationship with mean scores of attitude toward the level of HIV/AIDS awareness (*P*-value ≤ 0.05) (Table 5).

**Table 5:** Mean scores of knowledge of HIV/AIDS transmission modes, attitudes toward HIV/AIDS infected people, and attitudes toward the level of HIV/AIDS awareness.

**Variable**	***n***	**Knowledge of HIV/AIDS-transmission modes**		**Attitude toward HIV/AIDS-infected people**		**Attitude toward the level of HIV/AIDS awareness**	
		**Mean ±SD**	***P***	**Mean ±SD**	***P***	**Mean ±SD**	***P***
**Age (Year)**							
<20	145	84.1 ± 13.8	0.003	58.3 ± 14.5	0.000	58.1 ± 39.7	0.011
20–29	1,229	82.7 ± 15.4		52.4 ± 14.5		48.6 ± 42.2	
30–39	420	84.3 ± 13.1		52.7 ± 15		67.8 ± 39	
≥40	287	80 ± 15.7		53 ± 13.3		72.9 ± 37.2	
**Gender**							
Male	558	82.1 ± 15.3	0.241	53 ± 14	0.324	55.5 ± 39.4	0.637
Female	1,523	81.1 ± 17.3		54.1 ± 14.7		67.3 ± 41.1	
**Nationality**							
Saudi	1,963	82.4 ± 15.5	0.000	52.9 ± 14.3	0.314	62.1 ± 39.9	0.007
Non-Saudi	118	81 ± 15.7		53.8 ± 13.6		55.7 ± 39.9	
**Marital status**							
Single	1,187	68 ± 15.9	0.004	53.3 ± 14	0.103	65.8 ± 39.7	0.056
Married	817	67.5 ± 16.8		57.5 ± 17.5		31.3 ± 22.2	
Divorced	62	81.5 ± 16		45 ± 10.5		75.8 ± 10.2	
Widowed	15	87.8 ± 10.7		51.7 ± 14.9		53.6 ± 37.9	
**Education level**							
Uneducated	1	80.9 ± 15.6	0.000	52.8 ± 13.7	0.007	55.4 ± 38.2	0.001
Elementary	0	84.7 ± 15.5		53.6 ± 13.7		77.8 ± 33.8	
Intermediate	15	82.8 ± 15.7		53.9 ± 14.4		70 ± 39.8	
High school	317	85.8 ± 10.7		54 ± 13.4		77.8 ± 34.2	
Diploma	110	81.5 ± 15.3		52.5 ± 13.8		62.8 ± 40.8	
Bachelor	1,403	76.7 ± 17.5		50.1 ± 14.9		51.5 ± 38.8	
Higher diploma	23	77.9 ± 16.6		55.9 ± 14.7		61.2 ± 39.9	
Masters	154	85 ± 13.9		51.7 ± 14.3		60.3 ± 40.2	
PhD	54	83.6 ± 15.3		53.1 ± 14.5		52.3 ± 38.1	
**Monthly income (Saudi Arabian Riyal)**							
≤8,699	1,415	82.8 ± 14.7	0.002	50.9 ± 10.4	0.000	33.5 ± 37.3	0.213
8,700–11,999	266	85.7 ± 14		53.4 ± 14.2		33.8 ± 35.8	
12,000–15,299	179	81.9 ± 16.4		53.1 ± 13.9		63.9 ± 40.3	
15,300–20,159	137	81.1 ± 15		52.9 ± 14.8		76.9 ± 34.3	
≥20,160	84	78.1 ± 17.5		45 ± 10.5		51.5 ± 38.8	

A P-value of ≤ 0.05 is significant.

Spearman rank correlation explained significant negative linear correlations between knowledge of HIV/AIDS transmission modes-attitudes toward HIV/AIDS infected people and knowledge of HIV/AIDS transmission modes-attitudes toward the level of HIV/AIDS awareness of r = −0.040 and r = −0.070, respectively (*P-*value ≤ 0.05). Otherwise, there was a positive linear correlation between attitudes toward HIV/AIDS infected people-attitudes toward the level of HIV/AIDS awareness (r = 0.080, *p* = 0.072) (Table 6).

**Table 6:** Correlation between knowledge of HIV/AIDS transmission modes, attitudes toward HIV/AIDS infected people, and attitudes toward the level of HIV/AIDS awareness score.

**Variable**	**Correlation coefficient *r***	***P*** **-value**
Knowledge of HIV/AIDS transmission modes and attitudes toward HIV/AIDS infected people	−0.040	0.006
Knowledge of HIV/AIDS transmission modes and attitudes toward the level of HIV/AIDS awareness	−0.070	0.003
Attitudes toward HIV/AIDS infected people and attitudes toward the level of HIV/AIDS awareness	0.080	0.072

## Discussion

The present study provided insights on knowledge of HIV/AIDS transmission modes and attitudes toward HIV/AIDS infected people, as well as the level of HIV/AIDS awareness among the general population in the Kingdom of Saudi Arabia. The mean score of the participant's responses to knowledge items on HIV/AIDS transmission modes was 84.2 ± 15.8%.

Earlier surveys of the general population of Saudi Arabia (5, 27), Saudi medical school students (23, 28), Saudi non-medical school students (29), other countries in the region (30, 31), other African countries (32, 33) have reported a low level of understanding of HIV/AIDS. However, higher knowledge levels have been linked to younger age groups, better education, and knowing someone living with HIV/AIDS, implying that educational initiatives could positively influence the level of knowledge regarding HIV/AIDS. This has been demonstrated in European longitudinal surveys, where 15 years of ongoing teaching efforts greatly enhanced HIV and AIDS knowledge among high school students in Greece (34).

Inadequate understanding might lead to needless anxieties about interacting with HIV/AIDS patients, contributing to stigmatization (33). When the knowledge scores of males and females were compared, males had a higher knowledge score. Males have a greater HIV prevalence than females, which can be linked. During the decade 2000–2009, men accounted for two-thirds of all new cases (18). The gap in knowledge scores between male and female participants could also be linked to gender imbalance at the education level. This gap could also be related to differences in age profile in males and females. Accordingly, we recommend future studies to statistically illuminate these issues.

The mean score of the participant's responses to attitude items toward HIV/AIDS infected people was 50.1 ± 49.9%. The mean score of the participant's responses to attitude items toward the level of HIV/AIDS awareness in Saudi Arabia was 55.7 ± 3.6%. These findings came from the fact that a low percentage of the study respondents had positive attitudes toward HIV/AIDS infected people and the level of HIV/AIDS awareness among the general population in Saudi Arabia. Several other surveys in Saudi Arabia and other parts of the world have found similar negative attitudes, but at a lower level (5, 30–32, 35).

People living with HIV/AIDS were also stigmatized in this group. Treatment initiation has been demonstrated to be hampered by social stigma. Mahajan et al. examined the occurrence of societal stigma linked with HIV/AIDS and its detrimental influence on AIDS preventive actions, as well as measures for reducing the stigma (36).

In terms of HIV/AIDS preventive efforts, South Africa has established an interesting example in terms of community-based HIV awareness and education campaigns, HIV prevention research, and the introduction of antiretroviral medication (ART). This holistic strategy has resulted in enhanced community awareness, which has reduced social stigma and resulted in increased uptake of volunteer counseling and HIV testing (37).

HIV awareness has recently increased due to initiatives, marketing, and social media. Promoting an awareness campaign, such as the CDC's “Get Tested” initiative, which suggests that all people aged 13 to 64 be tested for HIV, would be a further step in enhancing routine health care (38). The need to reduce societal stigma around HIV/AIDS, provide social protection, and improve the general public's awareness and attitudes toward people living with HIV/AIDS have all been cited as essential aspects of the epidemic's control (35, 39, 40).

A comprehensive approach is needed to change those unfavorable views, including more support for people living with HIV/AIDS and the improvement of the healthcare system's many sectors. Patients' confidentiality must be ensured, as many Saudi Arabian patients still feel embarrassed seeking appropriate care, resulting in insufficient therapy and delayed treatment. Patients are often ignorant of the technological technologies employed in hospitals to provide anonymity, and there is a need to raise awareness of this. In response to the outbreak, the Saudi Ministry of Health launched a national AIDS program to monitor and coordinate efforts to prevent, diagnose, and treat HIV across the country. Every Saudi citizen infected with HIV or AIDS is entitled to free medical care and has their privacy about how they became infected protected by law. Another strategy for combating discrimination and stigmatization would be support groups.

The findings of the current study should be evaluated in light of several limitations. Though the sample is of reasonable size, the convenience sampling method was used, which has likely led to selection bias in our study population and the generalizability of our findings. The study was cross-sectional in design, limiting the making of causal inferences. There were no qualitative questions provided. A qualitative component could have aided in a better understanding of negative views about HIV/AIDS patients and provided a complete picture of the causes behind those sentiments.

## Conclusions

The study showed that a high percentage of the study respondents had good knowledge of HIV/AIDS transmission modes. However, a low percentage of the study respondents had positive attitudes toward HIV/AIDS infected people, and the level of HIV/AIDS awareness was also low. There were some statistically significant associations between several socio-demographic variables and mean scores of knowledge of HIV/AIDS transmission modes, mean scores of attitude toward HIV/AIDS-infected people, and mean scores of attitude toward the level of HIV/AIDS awareness. Significant negative linear correlations between knowledge of HIV/AIDS transmission modes-attitudes toward HIV/AIDS infected people, and knowledge of HIV/AIDS transmission modes-attitudes toward HIV/AIDS aware people were shown. Otherwise, there was a positive linear correlation between attitudes toward HIV/AIDS infected people-attitudes toward the level of HIV/AIDS awareness. Therefore, there is a dire need for advocacy campaigns and policies to help reduce HIV stigmatization.

## Data availability statement

The raw data supporting the conclusions of this article will be made available by the authors without undue reservation.

## Ethics statement

This study was reviewed and approved by the Faculty of Applied Medical Sciences ethics committee. Written informed consent was obtained from all participants for their participation in this study.

## Author contributions

SK and FQ had the idea for the paper, developed the conceptual approach, wrote the first draft, and revised the manuscript. RTA and SN conceptualized the study. RAA collected the data. MA and HM carried out statistical analyses. All authors have read and approved the final version of the manuscript.

## Conflict of interest

The authors declare that the research was conducted in the absence of any commercial or financial relationships that could be construed as a potential conflict of interest.

## Publisher's note

All claims expressed in this article are solely those of the authors and do not necessarily represent those of their affiliated organizations, or those of the publisher, the editors and the reviewers. Any product that may be evaluated in this article, or claim that may be made by its manufacturer, is not guaranteed or endorsed by the publisher.
